# A simple mathematical model using centred loops and random perturbations accurately reconstructs search patterns observed in desert ants

**DOI:** 10.1007/s00359-018-1297-6

**Published:** 2018-10-08

**Authors:** Franz Waldner, Tobias Merkle

**Affiliations:** 1 Physics-Institute, University of Zürich, Winterthurerstr. 190, 8057 Zürich, Switzerland; 2 Theoretical Biology, Faculty of Mathematics and Natural Sciences, University of Bonn, Kirschallee 1, 53115 Bonn, Germany; 3 Centre for Visual Sciences, Research School of Biology, The Australian National University, Canberra, ACT 2601 Australia

**Keywords:** Systematic search, Cataglyphis, Model, Loops, Stochastic element

## Abstract

This paper describes a new mathematical model that is based on centred loops to reconstruct the “Systematic Search” behaviour of *Cataglyphis* desert ants. The notable advantage of this model is the combination of simplicity, efficiency and performance. All model input is kept to a minimum, using only parameters that previous research has shown to be available to the animals at all times: distance from the origin, direction of the last step and home vector. Outbound and inbound search paths are being combined into loops that return to the origin, sampling this area more intensely. A stochastic element is added by random perturbations during the next step, mimicking unsystematic errors during the process of path integration and yielding the typical search patterns observed in *Cataglyphis* desert ants. The model output is compared to runs observed in the field.

## Introduction

In animal navigation, the process of path integration or dead-reckoning describes continuously measuring and summing up the details of every movement, thereby keeping a running total of distance to and direction of the starting point, known as the home vector (Mittelstaedt and Mittelstaedt 1973, 1980, 1982). Among numerous other species that have been studied extensively, desert ants have emerged as particularly astonishing navigators during their foraging trips (e.g. Wehner and Wehner 1986; Wehner et al. 1996; Wehner 2003, 2009).

While it only takes a simple mathematical model to recreate this particular ant behaviour (for reviews, see Benhamou and Séguinot 1995; Merkle et al. 2006b; Vickerstaff and Cheung 2010), these living “travelling integrators” are not as perfect as any model but much more flexible and adaptable. Their path integration is error-prone (Wehner and Wehner 1986; Müller and Wehner 1988; Merkle et al. 2006a) and errors have been shown to increase with increased duration of the foraging excursion (Merkle et al. 2006a; Merkle and Wehner 2010). Navigation by path integration alone might, therefore, not take an ant back to the nest entrance, which is often just a tiny hole in the desert ground. Hence, it seems imperative for them to have at least one backup system to avoid falling victim to heat, predation or desiccation. Desert ants of the genus *Cataglyphis* use, for instance, landmark-based orientation whenever available to improve their bearings (e.g. Collett et al. 1998; Wehner and Wehner 1986; Wehner 2003; see also Mangan and Webb 2012 for *C. velox*). Other cues that help them navigate are olfactory stimuli (Steck et al. 2009), surface structure (Merkle 2009), or tactile (Seidl and Wehner 2006) and magnetic cues (Fleischmann et al. 2018). These various systems are not thought to be switched on or off sequentially, but to operate in parallel at all times (Wehner et al. 2016). In the featureless desert terrain that many *Cataglyphis* species inhabit, with minimal visual landmarks and high volatility of odours, the so-called “Systematic Search” often represents the last resort to accurately locate the nest (Wehner and Srinivasan 1981; Müller and Wehner 1994; Merkle et al. 2006a).

The “Systematic Search” consists of patterns that have been described in great detail for desert ants of the genus *Cataglyphis* (e.g. Wehner and Srinivasan 1981; Müller and Wehner 1994; Merkle et al. 2006a; Merkle and Wehner 2009a, 2010) and the genus *Melophorus* (e.g. Schultheiss and Cheng 2011; Schultheiss et al. 2015), as well as other arthropods such as desert isopods *Hemilepistus* (e.g. Hoffman 1983a, b). The basic underlying routine of the search is as follows: the animal performs loops in different directions, always returning to the starting point before commencing a new loop, and expands the loops over time. This search pattern ensures that the area of the likeliest nest location (the starting point) is sampled most extensively while the overall search area gradually grows at the same time (Wehner and Srinivasan 1981). There is some flexibility in the routine, and studies have shown how the *Catalgyphis* search pattern is influenced, for instance by the length of the preceding foraging excursion: a longer foraging excursion inevitably causes larger errors in the path integrator, resulting in larger search patterns (Merkle et al. 2006a). Other influencing factors are the proximity to the nest entrance and familiarity of territory (Merkle and Wehner 2009a).

Ant navigation is clearly not all that straight-forward after all, and accurately representing the systematic search behaviour through mathematical modelling has been attempted for several decades now. Wehner and Srinivasan (1981) compared several *Cataglyphis* species and showed that the search function can be described as a radially symmetrical Gaussian curve, and they introduced simulations based on a probability density function (PDF). Mittelstaedt (1985) used loops without noise that were described by equations for the home vector. Müller and Wehner (1994) could reproduce the search patterns seen in *Cataglyphis fortis* by using a simple model where spirals regularly get intercepted by straight runs back to the starting point. Vickerstaff and Merkle (2012) incorporated integration errors in their Bayesian Model and also tested random search, spiral search and Lévy loop search, based on and compared against real search patterns of *C. fortis*.

In this paper, we present a simple “Systematic Search” model that uses centred loops, based on the home vector that can reproduce a number of observed search patterns in *C. fortis* desert ants. The model simulates the underlying biology and those unsystematic errors that add up in the animals’ path integration by introducing random noise to a rigid routine. We find that this technique can convincingly reconstruct the observed search loops.

## Materials and methods

### Why simulations?

The observation of animal behaviour presents us with the opportunity to reconstruct this behaviour using mathematical modelling. Each new model tests a specific idea about how the observed behaviour is being produced—or, more broadly, how the animal’s nervous system processes information and generates movement. This idea determines the underlying algorithm for the mathematical model with the aim to ultimately compare the model output to the observed behaviour and assess its performance and biological plausibility.

### How to develop equations that describe movements?

In a first step, the thorough analysis of the observed search patterns forms the basis of the model. Considering an otherwise featureless terrain, the only cues available to this model are the angle of the sun and the “odometer” (step counter), together computing the home vector. Every movement—along long and straight or rather short and more tortuous sections of the path or during the looping search pattern—has to be accounted for. A mathematical problem in this regard is the direction of the next step relative to the previous step, especially in loops that curve, during which the direction of every step likely deviates from the previous step.

### Which coordinate system?

A two-dimensional position can be described by two values (*x,y*), that is, by the *x*-coordinate and the *y*-coordinate in Cartesian space with fixed orthogonal directions *x* and *y*. The same position can also be described by the distance *r* from the origin and an angle *φ* relative to a reference direction fixed in space, known as azimuth in a polar coordinate system. In general, the home vector provided by the path integrator is described by the distance to and the direction of the origin. In contrast to the Cartesian system, the polar system has three peculiarities. First, to describe a movement from a (*r, φ*) position, two orthogonal directions are needed: parallel to the *r*-direction and parallel to the *φ*-direction, as well as orthogonal to the *r*-direction. As these directions are connected to each position, they are not fixed in space, but rather move with the position. Second, the units along the *φ*-direction are degrees of angles, different to the units of the distance; so the system has to deal with two unequal metrics. Third, the point of origin (0,0) cannot be used for computing the first movement since the *φ*-direction is not defined; an additional finite position (*r*_0_, *φ*_0_) is, therefore, needed.

### How to calculate the new position for an integration step?

Let us assume an ant is at position (*r,φ*) at time *t*, described by home vector u(*t*) from the origin. During time interval ∆*t*, the ant then moves a small integration step of length *s* in an arbitrary direction along the vector s, with *s* = │s│. Note that an integration step is not identical to a step of the ant. The new vector u(*t* + ∆*t*) is the vector sum u(*t*) + s. However, the new distance *r* and the new angle *φ* have to be evaluated by projecting the vector s onto the *r*-direction with component *s*_*r*_ and orthogonal with component *sφ* along the *φ*-direction. These two components together with *s* form a triangle with a right angle, thus *s*_*r*_^2^ + *sφ*^2^ = *s*^2^, but this result does not provide the respective lengths of the different components. To obtain those, knowledge of an angle of the triangle is essential. Let us now assume *γ* is the angle between the *r*-direction and the direction of the path vector s. The projected change of distance ∆*d*_║_ parallel to the *r*-direction is ∆*d*_║_ = ∆*r* = *s*_*r*_ = *s* cos(*γ*), the change of distance ∆*d*_┴_ in the orthogonal *φ*-direction is ∆*d*_┴_ =*s*_ *φ*_ = *s* sin(*γ*). Note that both values are in the units of distance. To transform to the units of angle, ∆*d*_┴_ has to be divided by *r*, thus ∆*φ* = ∆*d*_┴_/*r* = *s*_*φ*_/*r*, since ∆*d*_┴_ = ∆*φ r*, the distance ∆*d*_┴_ for a fixed angle ∆*ϕ* increases linearly with the radius *r*. These changes are added algebraically, including their sign, to the previous components *r*(*t*) and *φ*(*t*), thus increasing or decreasing the distance and increasing or decreasing the angle *φ* of the direction to the origin depending on whether the turning movement is clockwise or counter-clockwise.

### How to compare the directions of successive integration steps?

For the equations of movement, the change of angle between successive integration steps is needed. As the search patterns mainly consist of loops, a statistical analysis of the turning angle between the previous and the actual integration steps would result in a non-symmetric scatter. Disregarding the random deviations for now, the angle of two successive steps points to the same side. For simplicity, we assume that there is a constant increase by an incremental angle *β*. Hence, for the evaluation of the projections the angle *γ* increases by *β* at every following integration step with ∆*γ* = *γ*(*t* + ∆*t*) − *γ*(*t*) = *β*. Note that the reference *r*-direction of *γ* is also changing; therefore, the actual turning is a combination of these two changing angles.

### What do the equations of movement look like in detail?

The angle *γ* between the actual *r*-direction and the integration step vector s is changing according to:

1 $$\Delta \gamma =\gamma (t+\Delta t)-\gamma (t)=\beta ,$$

with constant incremental angle *β*. Note that smaller angles *β* give larger loops similar to larger length *s* of integration steps.

In polar coordinates, the *r*-direction for ∆*t* = 1 yields

2 $${s_r}=\Delta {d_{||}}=\Delta r=r(t+\Delta t)-r(t)=s\cos (\gamma ),$$

and the *φ*-direction, orthogonal to the *r*-direction, writes

3 $${s_\varphi }={\text{ }}\Delta {\text{d}} \bot =\Delta \varphi r=\{ \varphi (t+\Delta t) - \varphi (t)\} {\text{ }}r=s\sin (\gamma ).$$

The multiplication by r compensates for the unequal metric of polar coordinates. The turning angle ∆*φ* = ∆*d*_┴_/*r* is lager for small values of *r* (for equal ∆*d*_┴_). Hence close to the origin the turning is more rapid, as the length of integration step *s* is kept constant.

The above routine (using MATLAB version 4.2b) results in centred loops spreading in different directions. However, over time the loops start to drift away from the centre. Therefore, an additional factor that reduces the turning angle when approaching the centre needs to be introduced. This is achieved by changing the length of the integration steps when approaching the centre: the term of │cos(*γ*)│ *b* gets subtracted (*b* < 1), resulting in straighter paths. Hence,

4 $${s_r}=\Delta r=r(t+\Delta t)-r(t)=s\{ \cos (\gamma ) - \left| {\cos (\gamma )} \right|b\} .$$

When approaching, a negative cos(*γ*) = − *a* results in − *a*(1 + *b*), thus leading to a larger reduction of the distance *r* and a smaller turning angle. Instead, when leaving, a positive cos(*γ*) = + *a* results in + *a*(1–*b*); the increase of r is, therefore, smaller and the turning angle larger. Note that only paths are calculated, not speeds.

### How do the equations transform into Cartesian (*x,y*) coordinates?

The angular variation remains the same in this frame:

5 $$\Delta \gamma =\gamma (t+\Delta t)-\gamma (t)=\beta ,$$

with incremental angle *β*.

The polar directions are transformed into a vector u, with components (*x,y*) with absolute value *u* = │u│ =  √(*x*^2^ + *y*^2^).

The unit vector e_*r*_ is along the *r*-direction, thus parallel to the direction of u, with components:

6 $${\underline {{\text{e}}} _r}(x)=x/u \quad {\text{and}} \quad {\underline {{\text{e}}} _r}(y)=y/u.$$

The unit vector e_*φ*_ is along the *φ*-direction, orthogonal to the direction of u, with components:

7 $${\underline {{\text{e}}} _\varphi }(x)=-\,y/u \quad {\text{and}} \quad {\underline {{\text{e}}} _\varphi }(y)=x/u.$$

The equations of motions in the Cartesian frame read:

8 $${\underline {{\text{s}}} _r}(x)=s\{ \cos (\gamma ) - \left| {\cos (\gamma )} \right|b\} (x/u),$$

9 $${\underline {{\text{s}}} _r}(y)=s\{ \cos (\gamma ) - \left| {\cos (\gamma )} \right|b\} (y/u),$$

for the *r*-direction and

10 $${\underline {{\text{s}}} _\varphi }(x)=s\sin (\gamma )(-\,y/u),$$

11 $${\underline {{\text{s}}} _\varphi }(y)=s\sin (\gamma )(x/u),$$

for the *φ*-direction.

Finally, the new vector u(*t* + ∆*t*) after time ∆*t* consists of the two components:

12 $${\underline {{\text{u}}} _x}(t+\Delta t)={\underline {{\text{u}}} _x}(t)+{\underline {{\text{s}}} _r}(x)+{\underline {{\text{s}}} _\varphi }(x),$$

13 $${\underline {{\text{u}}} _y}(t+\Delta t)={\underline {{\text{u}}} _y}(t)+{\underline {{\text{s}}} _r}(y)+{\underline {{\text{s}}} _\varphi }(y).$$

Even in the (*x,y*) frame, starting at (0,0) is not possible; hence, a small finite vector (*x*_0_,*y*_0_) replaces the initial polar vector (*r*_0_, *φ*_0_). Further, the initial angle *γ*_0_ is set to *α*, hence *γ*_0_ = *α*. After setting the initial angle, the incremental angle *β* and the parameters *s *(size) and *b* (backward), as well as setting the initial conditions (*x*_0_,*y*_0_) for u(*t* = 0), it is straight-forward to evaluate the noiseless structure by programming a mathematical loop with the above Eqs. (5–13) and plotting the resulting patterns.

### How to introduce random perturbations?

To reconstruct the observed search patterns, random perturbations are introduced. They are defined by two parameters: a random perturbation factor *f* and a random number *ψ*. Since the observed patterns rarely feature sharp turns, instead their curvature is fairly smooth, the random perturbation *fψ* is not applied at every integration step. During *n* integration steps, only two normalised random numbers *fψ* are generated. These random numbers have a normal distribution with mean 0 and variance 1. Each command rand(‘normal’) generates a positive or negative random number *ψ*_*k*_ of which the first is used for the *x*-component, multiplied by the factor *f*. At the same integration step, the second rand(‘normal’) creates a new independent number *ψ*_*k*+1_, again multiplied by *f*, for the *y*-component. Hence, a random vector p is formed with components (*fψ*_*k*_, *fψ*_*k*+1_). During *n* integration steps, the same random vector p is added to the vector u, first with increasing, then with decreasing magnitude, to create vectors u′:

14 $${\underline {{\text{u}}} ^\prime }_{x}={\underline {{\text{u}}} _x}+\sin ({\sigma _i})f{\psi _k},$$

15 $${\underline {{\text{u}}} ^\prime }_{y}={u_y}+\sin ({\sigma _i})f{\psi _{k+1}},$$

with

16 $$\sigma =i\pi /(n+1),$$

with integer i ranging from 1 to *n*.

This random vector p might point in a very different direction relative to the noiseless position vector u. Therefore, the new path u′ could turn less, leading to a larger loop, or turn more, giving a smaller loop. Hence, even with noiseless loops u of equal size, randomness produces different magnitudes of loops, without the need to vary the parameters *s* or *β* during one run.

In order to reduce sharp turns caused by the finite integration steps, the simulated patterns are averages of five successive steps. The runs are now repeated including randomness (Eqs. 14–16), and plotted together with the distance of the positions relative to the origin. Each run produces a unique pattern, since the sequence of random numbers is highly unlikely ever the same.

### How do errors affect integration?

Finally, the ants have to cope with errors during their integration of the search path. It is not clear how the ants’ integration system responds to individual unsystematic errors (random perturbation) or if only a “critical mass” of errors triggers a response. On average, random deviations to the left and to the right cancel each other out, although never fully, given the limited number of random numbers per loop. Further, the average of absolute random deviations increases with the square root of time (Einstein 1905), resulting in larger loops.

### Which parameters make up the model?

The model is governed by eight parameters: initial angle *α*, incremental angle *β*, backward factor *b*, integration step length *s*, random perturbation factor *f*, number *n* of steps to activate random perturbation, and finite initial conditions (*x*_0_,*y*_0_) in *x*- and *y*-direction. However, most of these values stay the same for each run, and *n* is only changed for fine-tuning. Further, both increasing the integration step size *s* and decreasing the incremental angle *β* result in an increased loop size. Hence, the parameters that define the shape of the search loops most are *b, s*, and *f*.

First, the influence of parameters *b, s, f*, is discussed and then tested under various assumptions. Then, the model is used to simulate search patterns of *C. fortis*.

For simplicity, the chosen parameters are constant for each run. However, we acknowledge that increasing the step length or decreasing the incremental angle for longer trips might be plausible to account for the growing area covered by the search patterns.

After setting all values, the algorithm continuously creates search patterns.

### How do results compare to observed search patterns?

The nine search patterns of *C. fortis* desert ants used for comparison with the present model were reproduced from Vickerstaff and Merkle (2012) and had originally been recorded by T. Merkle and published by Merkle and Wehner (2008). They were selected for modelling after rigorous analysis and scrutiny of suitability (see Vickerstaff and Merkle 2012 for details). All search runs were recorded for 300 s. For each search run, the distance to the origin over time is presented as well.

## Results

### Variation of parameters

**Fig. 1:**
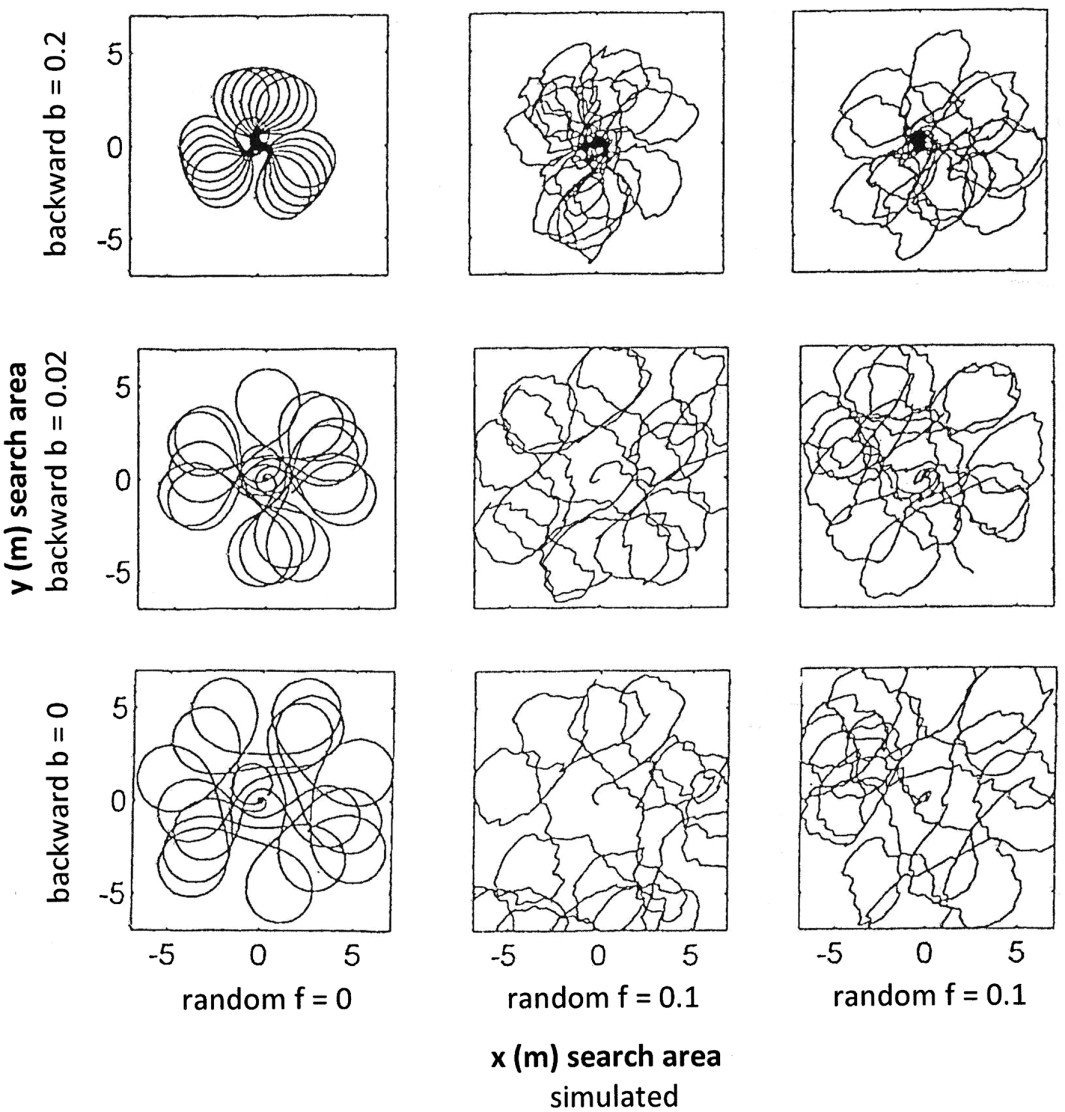
Set of simulations with no random perturbation *f* = 0 in one model run (left column) and random perturbation factor *f* = 0.1 in two model runs (centre and right columns). Other model parameters: initial angle *α* = 10, incremental angle *β* = 3, integration step length *s* = 0.12, number of steps to activate random perturbation *n* = 3. The variable component is backward factor *b*, set to: *b* = 0.2 (top row), *b* = 0.02 (centre row), and *b* = 0 (bottom row). Paths do not lead back to the origin if backward parameter is absent or weak

**Fig. 2:**
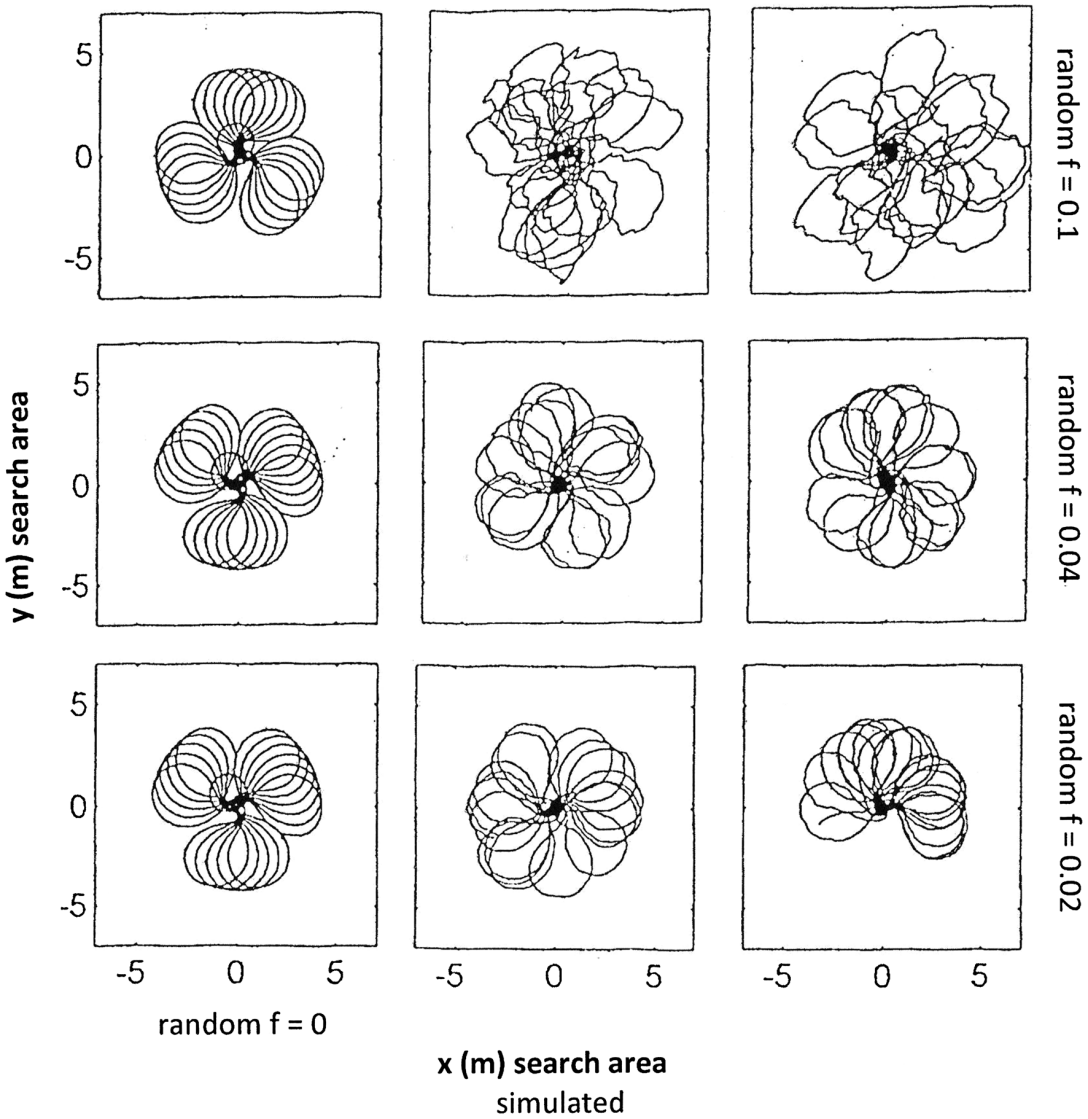
As Fig. 1 but the variable component is the random perturbation factor *f* (centre and right columns; left column: no random perturbation). Top row: *f* = 0.1, centre row: *f* = 0.04, bottom row *f* = 0.02. Only the strongest perturbation *f* = 0.1 causes the loops to break

**Fig. 3:**
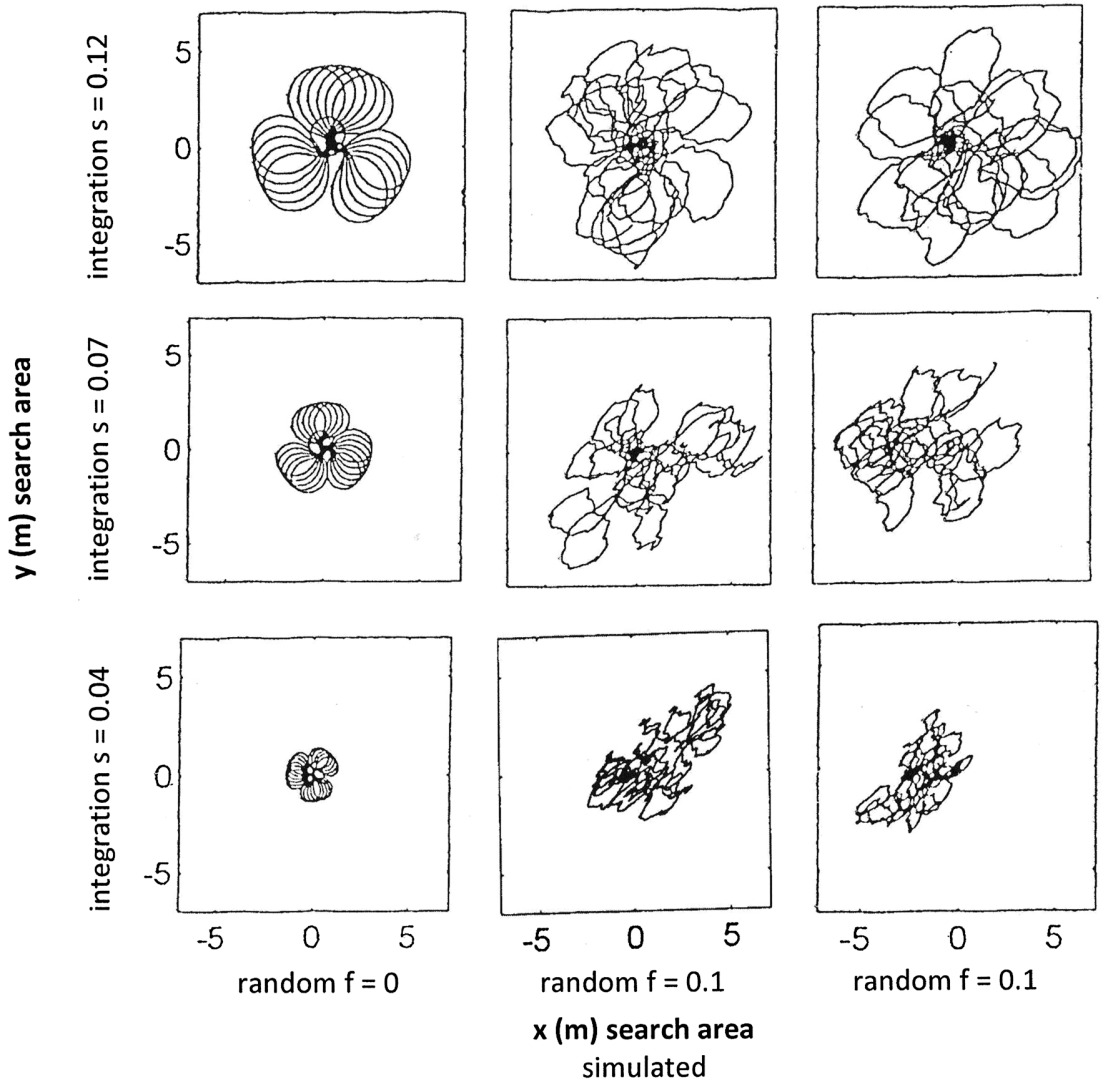
As Fig. 1 but the variable component is integration step length *s*, set to: *s* = 0.12 (top row), *s* = 0.07 (centre row), and *s* = 0.04 (bottom row). Search patterns are confined to much smaller areas when steps are shorter

We first varied the three main model parameters “backward factor b”, “random perturbation *f*” and “step length *s*”—presented in rows in Figs. 1, 2 and 3, respectively.

In all three figures,

- The first row is identical in that the model used the following parameters: initial angle *α* = 10, incremental angle *β* = 3, integration step length *s* = 0.12 and backward factor *b* = 0.2.
- The left column presents the model output in the absence of random perturbation (*f* = 0), while the centre and left columns depict the model output with the effect of random perturbation *f*. These two runs had perturbation factor *f* = 0.1 and identical parameter settings, except that the specific random numbers *ψ*_*k*_ (within *fψ*_*k*_) are truly random, i.e. most likely different for each run.

The perturbation factor *f* is set to *f* = 0.1 in all rows of Figs. 1 and 3, but is different in every row in Fig. 2.

Figure 1: effect of backward factor *b*.

The backward factor *b* was decreased from *b* = 0.2 to *b* = 0.02 and to *b* = 0 (rows, respectively). Without any random perturbation (left column), a larger *b* produces a search pattern that approaches the origin recurrently, whereas a smaller *b* causes the path to stay clear of the origin. This is also true for searches with random perturbation (*f* = 0.1, centre and right columns); the difference being that unperturbed runs are much smoother. For backward action *b* = 0, the model does not reproduce the typical “clover leaf” pattern anymore; rather a “spinning top” pattern in that the search path stays far away from the origin, indicating the role of factor *b* in leading the ants home.

Figure 2: effect of random perturbation factor *f*.

The random perturbation factor *f* was decreased from *f* = 0.1 to *f* = 0.04 and to *f* = 0.02 (rows, respectively; also centre and right columns). A small *f* (bottom row) results in a slight scatter of the loops. An increase in *f* (centre) improves the patterns, although the shape of the loops remains very similar. Only a substantially larger *f* (top row**)** is able to produce larger and smaller loops although the noiseless loops have all the same size.

Figure 3: effect of step length *s*.

The integration step length *s* was decreased from *s* = 0.12 to *s* = 0.07 and to *s* = 0.04 (rows, respectively). The size of the search loops shrinks or grows with smaller or larger integration steps, respectively, while the overall pattern stays the same. Paths return to the origin in both unperturbed (left column) and perturbed scenarios (centre and right columns).

### Simulated and observed search patterns

**Fig. 4:**
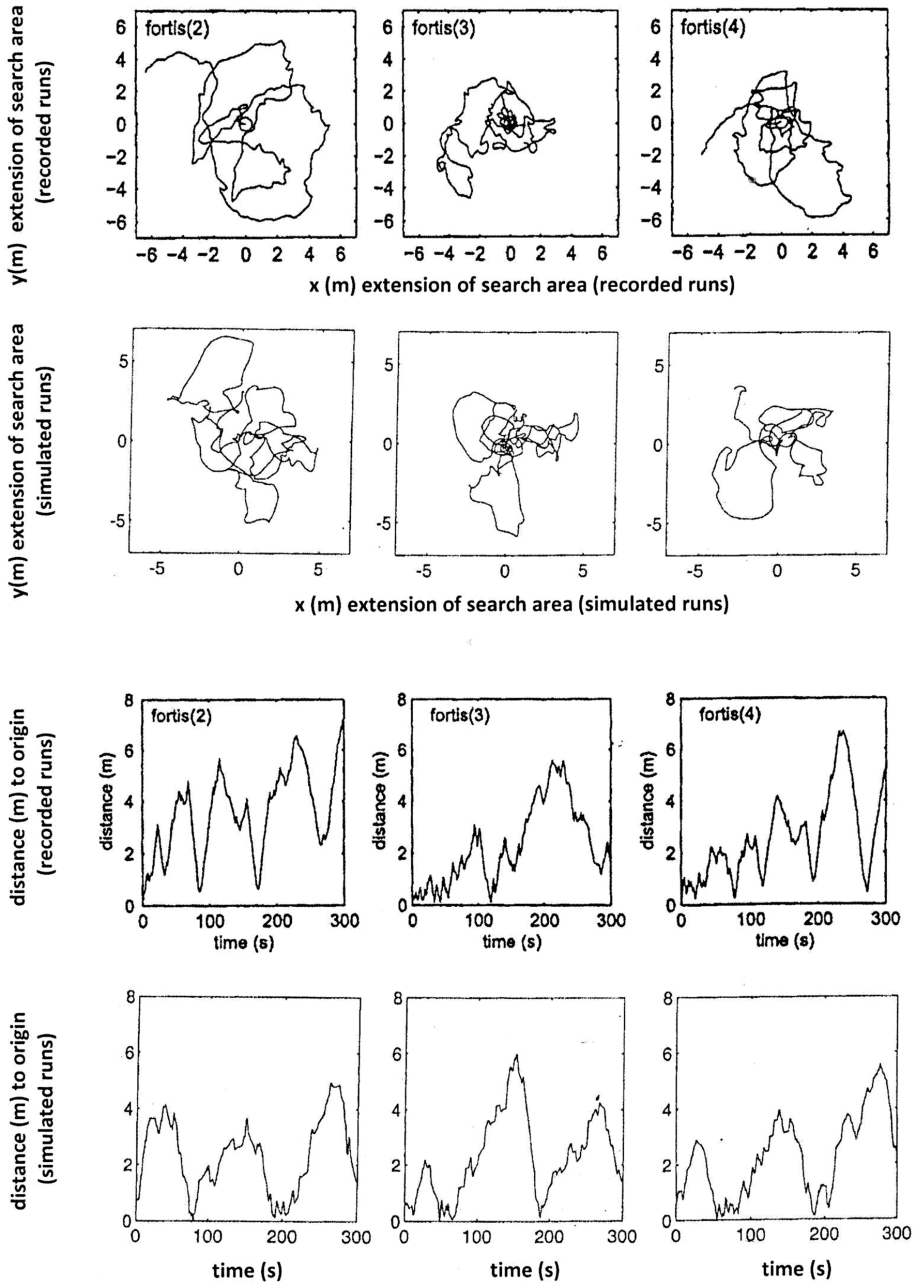
Three observed search patterns of *Cataglyphis fortis* (top row), compared to simulated search patterns (second row), and measured distances to the origin over the duration of the recorded and simulated trips (third and fourth row). The recorded patterns and the measured distances of the recorded trips were reproduced and adapted with permission from Vickerstaff and Merkle (2012)

**Fig. 5:**
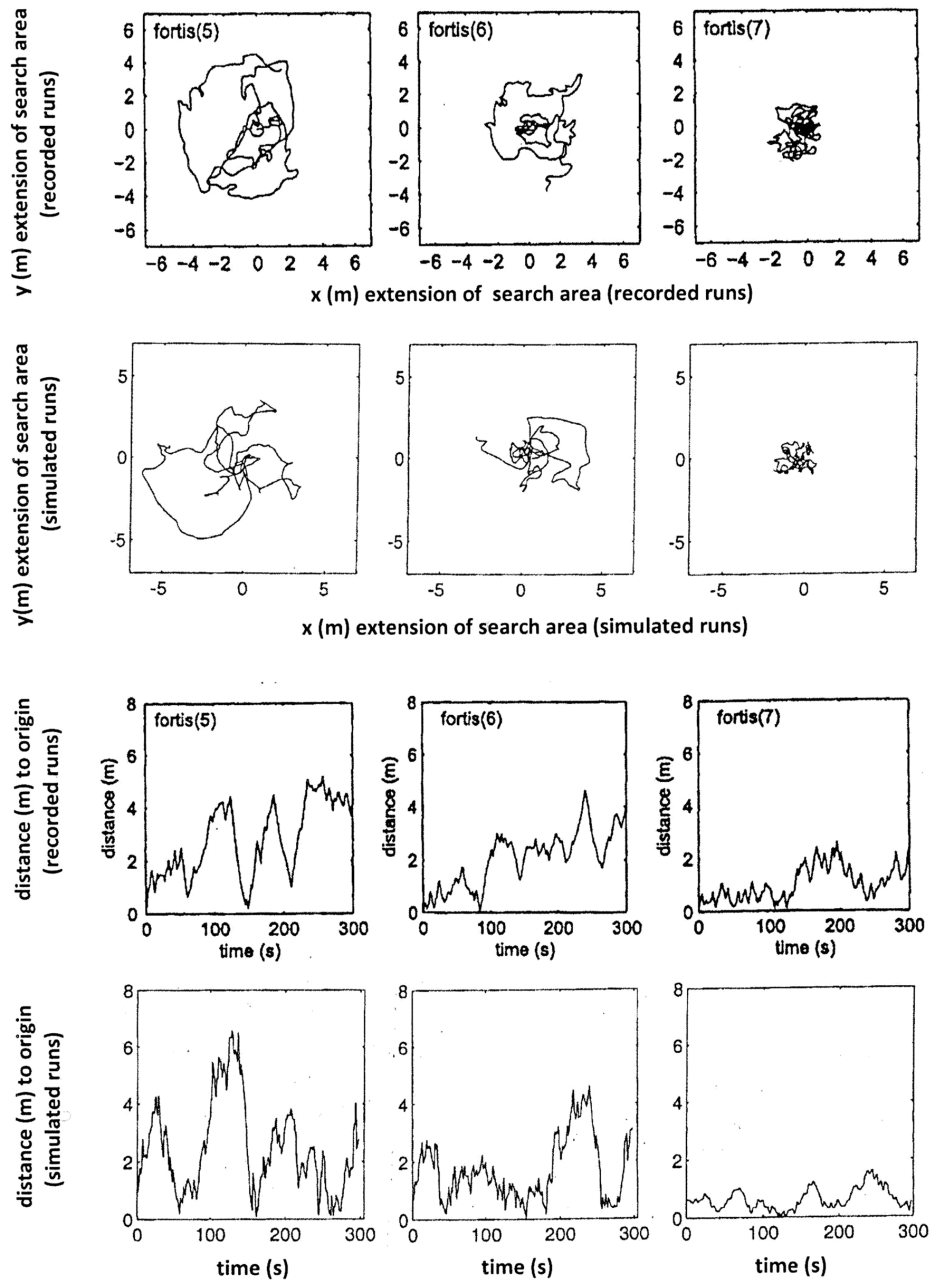
As Fig. 4 but for another three *C. fortis* search patterns

**Fig. 6:**
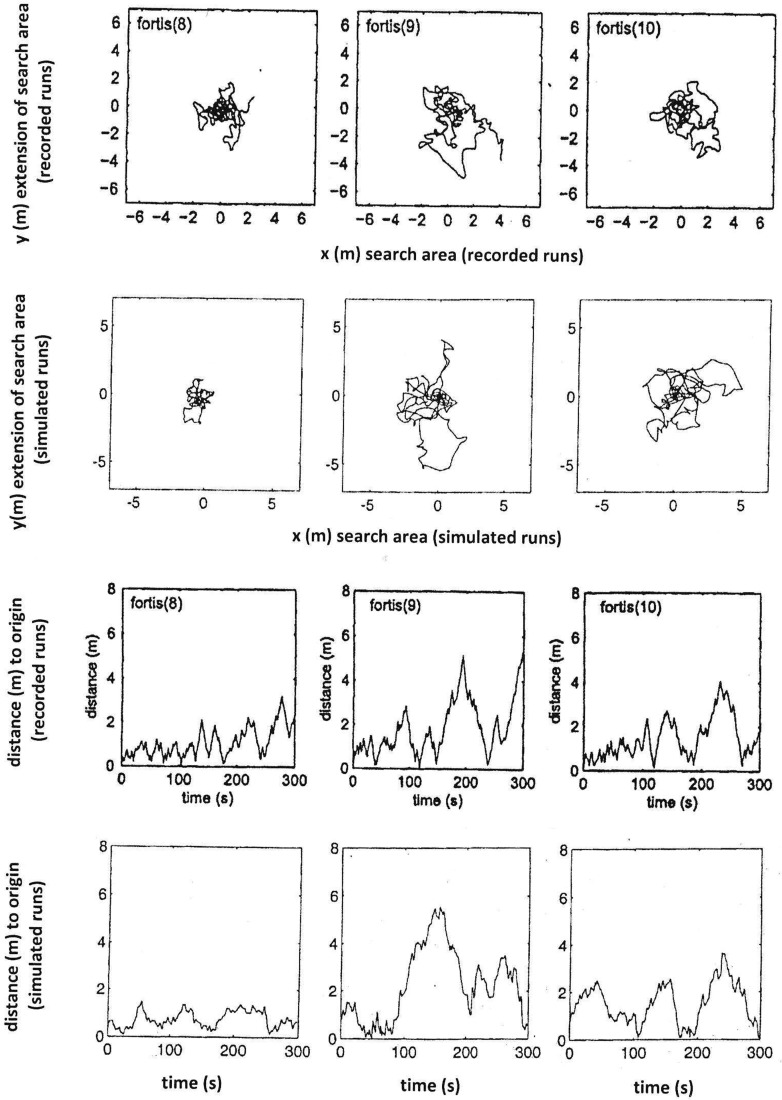
As Fig. 4 but for another three *C. fortis* search patterns

The performance of the model is assessed by its ability to reproduce the characteristics of typical search patterns. In programming this model, parameters were adjusted after initial runs to minimise discrepancies between model output and observed search patterns. Keeping in mind that any individual ant would exhibit different search patterns every single time as well, the two identical model runs (centre and right columns in Figs. 1, 2, 3) illustrate how the stochastic effect can produce qualitatively equivalent paths that differ in their detail. The best fit for each run is shown in Figs. 4, 5 and 6 for nine recorded search runs of *C. fortis*: recorded search patterns of *C. fortis* (Vickerstaff and Merkle 2012) are compared to simulated search patterns (see Table 1 for parameters), and measured distance to the origin over the duration of the recorded and simulated trips are presented.

**Table 1:** Parameters of the simulations used for Figs. 4, 5 and 6: initial angle *α*, incremental angle *β*, integration step length *s*, backward factor *b*, random perturbation factor *f*, number of steps to activate random perturbation *n*

Number *C. fortis*	(2)	(3)	(4)	(5)	(6)	(7)	(8)	(9)	(10)
*α*	10	10	10	10	10	10	10	10	10
*β*	3	3	3	3.5	3.5	3	3	3	3
*s*	0.08	0.08	0.1	0.15	0.15	0.018	0.003	0.08	0.08
*b*	0.3	0.3	0.3	0.3	0.2	0.3	0.3	0.3	0.3
*f*	0.2	0.2	0.2	0.6	0.4	0.1	0.1	0.2	0.2
*n*	3	3	3	2	2	3	3	3	3

Overall, our simple model is capable of reproducing the search paths of *Cataglyphis* desert ants and, in particular, the crucial “Systematic Search” component of recurrently returning to the origin.

## Discussion

### Required input information

The model presented here is based on the animal’s home vector. Animals that navigate by path integration have the home vector available at all times during foraging; that is, it gets updated continuously. A second requirement for the plausibility of our model is that the animal has an efference copy, the direction of the preceding integration step. This direction, defined as angle *γ* relative to the home vector, is essential to keep the home vector up to date. During its next integration step, the animal will head in a different direction while still turning to the same side (again, relative to the home vector) within one particular loop, but with an (by angle *β*) increased *γ*. Search patterns will frequently be influenced by random deviations, but nonetheless is the animal still capable of maintaining the main structure of its loops simply by turning to one side more often or more strongly, while of course still integrating the direction and distance to the centre. Hence, one of the main advantages of our model is its simplicity: all information that the animal needed in order to perform this kind of search behaviour are length and angle of the home vector, plus the direction of the last step. Our model does not require the animal to have a memory of previous paths but solely draws on information to successfully perform path integration in the first place. Simply put, the direction of the last step updates the path integrator and also flows into determining the next step.

Admittedly, simplicity alone does not necessarily favour a particular theoretical approach, and simple mathematical formulae might not per se lead to biologically plausible representations (Maurer and Séguinot 1995). However, it is certainly undisputed that desert ants can only make use of limited memory capacities (Bélisle and Cresswell 1997; Dukas 1999), even if this means sacrificing a potentially improved performance. For instance, they only remember the distance of the most recent outbound run (Cheng et al. 2006) and do not improve the accuracy of their path integrator after repeated, identical trips (Merkle and Wehner 2009b). Also, they only use the distance available through the path integrator, rather than the overall length of the preceding foraging excursion for adjustments in the extension of their search patterns (Merkle and Wehner 2010). In the light of these examples, noting how efficiently *Cataglyphis* ants appear to use their memory capacities, a “Systematic Search” routine that only requires two readily available values has obvious merits.

### Comparisons with previous simulations

In their comprehensive study of search behaviour in *Cataglyphis* ants, Wehner and Srinivasan (1981) showed recorded search pattern and presented the distance r to the origin as a function of the overall path length for four individual ants. These patterns differ in their frequency and magnitude of oscillation. The result of their simulations were loops that regularly return to the origin but spread out into different directions and increase their distance to the origin over time. In general, their simulations resembled the recorded patterns fairly well. Lacking the rapid random deviations of real foraging excursions though, their simulated paths appeared much smoother. The *r*/*t* diagrams all had very similar shapes, the simulated paths always returned exactly to the origin and continuously increased over time. This resulted from the implementation of the idea of a posterior probability distribution function PDF for the variation of r, based on the memory of all the past performances, and lacking randomness.

**Fig. 7:**
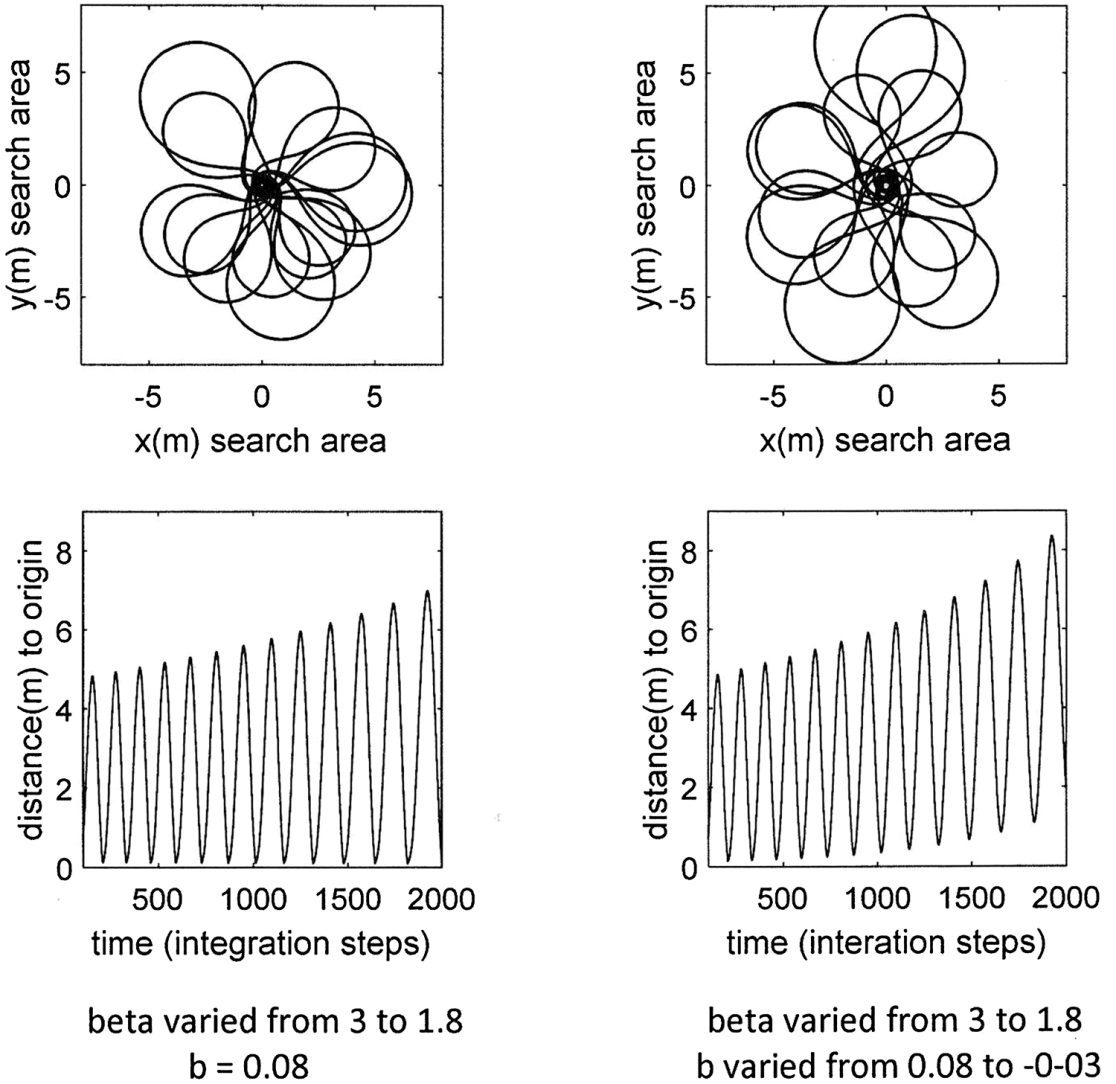
Continuous variation of selected parameters during a simulation. Initial angle *α* = 10 and integration step length *s* = 0.12 are constant and no perturbation was implemented (*f* = 0). Left and right columns: the incremental angle *β* decreases continuously from *β* = 3 to *β* = 1.8. Right column: backward factor *b* also decreases from *b* = 0.08 to *b* = − 0.03

Our present model applies rapid random deviations. More importantly, the *r*/*t* function shows a structure that is more similar to the recorded search paths, except that they return to the origin more often and the loops grow less over time. Both discrepancies can easily be minimised by reducing the incremental angle *β* (higher maxima) and the backward factor *b* (higher minima) in the later stages of the search. The effects of these modifications are shown in Fig. 7.

In the model by Müller and Wehner (1994), the simulated animal commenced a spiral to which jitter was added. The spiral search was reset whenever the home vector reached a critical length as a function of path length. Then, the angle between the animal’s walking direction and the home vector was reduced, causing the animal to walk back towards the origin before spiralling out again. The simulated patterns resembled the recorded paths to a degree but had distinct sharp corners.

Our present model shares similarities with Müller and Wehner (1994), with the exception that we do not model the spiralling out and returning home as two separate phases, but rather we combine them into a loop. This loop is defined and executed by continuously varying the vector between walking direction and direction of the home vector and by adding a simple term to approach the origin.

Vickerstaff and Merkle (2012) showed that a heuristic Bayesian search with the aim to maximise the probability of finding the nest resembles the search patterns of *C. fortis* very well. Their model outperformed three simpler approaches but the authors admitted that their model was using a fairly costly, high-resolution spatial PDF and might, therefore, not necessarily be representative of a miniature insect brain. As discussed above, our present model is more economic.

## Conclusions

Centred loops, at the core of our model, combine mathematical simplicity and performance.

The model creates first loops that return back to the starting point, resulting in a higher search density around the point of origin. Random perturbations are being applied successively, producing search patterns that match those of desert ants *C. fortis*. Search paths are curved to form loops, both on their way out and as they return back to the origin, improving the search in efficiency and leading the animals back to the nest as quickly as possible. Here, we used search paths of ants that were not biased towards any particular direction i.e. the path integrator had been reset to zero and errors had been nulled before starting their searches (see Vickerstaff and Merkle 2012). The next step would be to apply this model to ants that have not reset their path integrator and to test for an effect of preceding foraging excursions.
